# Baseline neutrophil-to-lymphocyte ratio and c-reactive protein predict efficacy of treatment with bevacizumab plus paclitaxel for locally advanced or metastatic breast cancer

**DOI:** 10.18632/oncotarget.27423

**Published:** 2020-01-07

**Authors:** Yoshimasa Miyagawa, Ayako Yanai, Takehiro Yanagawa, Junichi Inatome, Chiyomi Egawa, Arisa Nishimukai, Kaori Takamoto, Takashi Morimoto, Yuichiro Kikawa, Hirofumi Suwa, Tomoe Taji, Ai Yamaguchi, Yuki Okada, Atsushi Sata, Reiko Fukui, Ayako Bun, Hiromi Ozawa, Tomoko Higuchi, Yukie Fujimoto, Michiko Imamura, Yasuo Miyoshi

**Affiliations:** ^1^ Department of Surgery, Division of Breast and Endocrine Surgery, Hyogo College of Medicine, Nishinomiya, Hyogo 663-8501, Japan; ^2^ Department of Surgery, Kansai Rosai Hospital, Amagasaki, Hyogo 660-8511, Japan; ^3^ Department of Breast Surgery, Yao Municipal Hospital, Yao, Osaka 581-0069, Japan; ^4^ Department of Breast Surgery, Kobe City Medical Center General Hospital, Chuo-ku, Kobe, Hyogo 650-0047, Japan; ^5^ Department of Breast Surgery, Hyogo Prefectural Amagasaki General Medical Center, Amagasaki, Hyogo 660-8550, Japan

**Keywords:** breast cancer, bevacizumab, neutrophil-to-lymphocyte ratio, c-reactive protein, predictive marker

## Abstract

The effect of bevacizumab plus paclitaxel therapy on progression-free survival (PFS) is prominent; however, no overall survival (OS) benefit has been demonstrated. Our aim was to study the predictive efficacy of peripheral immune-related parameters, neutrophil-to-lymphocyte ratio (NLR), absolute lymphocyte count (ALC), and c-reactive protein (CRP) in locally advanced and metastatic breast cancers. A total of 179 patients treated with bevacizumab plus paclitaxel were recruited from three institutes in the test cohort. The cut-off values of NLR, ALC, and CRP were set at 3, 1500/μL, and 1.0 mg/dL, respectively, and baseline values of these factors were measured. The PFS of patients with NLR-low was significantly longer than that of patients with -high (median, 12.6 vs. 7.2 months; hazard ratio (HR), 0.48, 95% confidence interval (95% CI), 0.31–0.73; *p* = 0.0004). OS of patients with NLR-low was significantly better than those with-high (22.2 vs. 13.5 months; HR, 0.57, 95% CI, 0.39–0.83; *p* = 0.0032). Similarly, improved PFS and OS were recognized in patients with CRP-low as compared with patients with -high (HR, 0.44, 95% CI, 0.28–0.68; *p* = 0.0001 and HR, 0.39, 95% CI, 0.26–0.61, *p* < 0.0001, respectively). In the validation cohort from two institutes (*n* = 57), similar significant improvements in PFS and OS were confirmed for patients with NLR-low (*p* = 0.0344 and *p* = 0.0233, respectively) and CRP-low groups (*p* < 0.0001 and *p* = 0.0001, respectively). Low levels of NLR and CRP at baseline were significantly associated with improved prognosis in patients treated with bevacizumab plus paclitaxel.

## INTRODUCTION

It has been well established that angiogenesis is a critical process in progression of not only early breast cancer but also metastatic breast cancer (MBC) [1]. Vascular endothelial growth factors (VEGFs) are key players of angiogenesis, that are generated by the action of VEGFs through stimulating proliferation and migration of vascular endothelial cells [2]. Angiogenesis accompanies altered extracellular matrix and increased permeability of neovasculature, resulting in limited efficacy of chemotherapy due to decreased delivery of drugs and therapeutic agents into tumor cells [3]. Inhibition of angiogenesis with chemotherapy, therefore, is an ideal strategy for cancer treatment, not only with direct inhibition of blood supply, but also by improvement of drug delivery.

Bevacizumab, a humanized monoclonal antibody, is an inhibitor directed against VEGF-A, and blocks binding of VEGF-A to receptors including vascular endothelial growth factor receptor (VEGFR)-1, VEGFR-2, and neuropilin-1 [4]. A randomized phase III trial on 722 patients (E2100 trial) with metastatic breast cancer not previously treated with chemotherapy demonstrated improvement in progression-free survival (PFS) with the addition of bevacizumab to paclitaxel, as compared with paclitaxel alone (hazard ratio (HR), 0.60; 95% confidence interval (CI), 0.51–0.70; *p <* 0.001; median PFS, 11.8 vs. 5.9 months) [5]. In contrast to longer PFS, no improvement to overall survival (OS) of patients administered bevacizumab was reported (HR, 0.88; *p =* 0.16; 26.7 vs. 25.2 months). Similarly, addition of 15 mg/kg bevacizumab to docetaxel treatment improved PFS (HR, 0.77; 95% CI, 0.64–0.93; *p =* 0.006; 10.1 vs. 8.2 months), but not OS (HR, 1.03; 95% CI, 0.7–1.33; *p =* 0.85; 30.2 vs. 31.9 months) as compared to treatment with docetaxel alone as first-line therapy for MBC in a phase III AVADO trial [6]. In a randomized phase III trial (*n =* 1237 cases), which compared chemotherapy (capecitabine, taxane-based, or anthracycline-based chemotherapy) plus placebo with chemotherapy plus bevacizumab (RIBBON-1), PFS was longer for the bevacizumab group (HR, 0.69; 95% CI, 0.56–0.84; *p <* 0.001; median PFS, 5.7 vs. 8.6 months for capecitabine; HR, 0.64; 95% CI, 0.52–0.80; *p <* 0.001; median PFS, 8.0 vs. 9.2 months for taxane and anthracycline) [7]. However, no statistically significant differences in OS were reported between treatments with and without bevacizumab. This situation was further confirmed in a meta-analysis consisting of 2447 patients, which demonstrated no significant difference in OS (HR, 0.97; 95% CI, 0.86–1.08; median OS, 26.7 vs. 26.4 months), although significant improvement in PFS of patients treated with bevacizumab was obtained (HR, 0.64; 95% CI, 0.57–0.71; median PFS 9.2 vs. 6.7 months) [8]. Therefore, development of a robust biomarker that predicts benefit of bevacizumab in terms of OS, is a critical issue in daily clinical practice.

Superior PFS induced by bevacizumab is consistently recognized, irrespective of subgroups based on clinical factors [5–7]. Since bevacizumab acts through abrogation of VEGF-A, its blood levels or factors that influence VEGF-A activity seem to be associated with bevacizumab efficacy. Therefore, circulating levels of short VEGF-A isoforms or VEGF-A receptors (neuropilin-1 and VEGFRs) in tumors or plasma are emphasized as candidates for bevacizumab biomarkers [9]. In the AVADO trial, biomarker analyses using plasma proteins, blood mRNA levels, immunohistochemistry of tumor tissue, and single-nucleotide polymorphisms of VEGF pathway-related genes in germline DNA were reported [10]. Of the biomarkers analyzed, VEGF-A and VEGFR-2 were identified as potential predictors for bevacizumab efficacy. On the basis of these results, VEGF-A was evaluated in a phase III trial (MERiDiAN), which compared first-line therapy for human epidermal growth factor receptor 2 (HER2)-negative MBC in 481 patients between paclitaxel plus bevacizumab and paclitaxel plus placebo groups, with prospective biomarker evaluation [11]. In this study, plasma VEGF-A (pVEGF-A) was measured and randomized according to baseline pVEGF-A concentration (< 5.05 vs. ≥ 5.05 pg/mL) into paclitaxel plus bevacizumab or placebo. The HRs of bevacizumab were 0.68 (99% CI, 0.51–0.91; *p =* 0.0007) in the intent-to-treat population and 0.64 (96% CI, 0.47–0.88; *p =* 0.0038) in the pVEGF-A high subgroup, respectively. Since the PFS by treatment-by-VEGF-A interaction was not statistically significant (*p* value for interaction, 0.4619), pVEGF-A was concluded to not be a predictive marker for addition of bevacizumab [12].

Recently, direct and indirect effects of VEGF on immune-related cells have been focused on. VEGF modulates the various functions of cancer immunity involving promoting regulatory T cells (Tregs), inhibition of dendritic cell (DC) maturation, stimulation of differentiation to tumor-associated macrophages (TAMs), and infiltration of myeloid-derived suppressor cells (MDSCs), leading to an immune suppressive microenvironment in the tumor [13]. Since bevacizumab inhibits VEGF activity, bevacizumab seems to involve amelioration of the immune microenvironment, mediated by inhibiting VEGF actions on immune-related cells, as described above. If this speculation is true, local or systemic immunity against breast cancer seems to be associated with bevacizumab efficacy. Since we demonstrated that a peripheral immune-related biomarker, neutrophil-to-lymphocyte ratio (NLR), is a predictor for eribulin [14], predictive significance of NLR and absolute lymphocyte count (ALC) for bevacizumab efficacy were investigated in patients treated with bevacizumab plus paclitaxel for locally advanced and metastatic breast cancer. In addition, a peripheral inflammatory biomarker, c-reactive protein (CRP) was also evaluated.

## RESULTS

### Determination of optimal cut-off values of NLR and CRP

In order to identify the best cut-off values for NLR and CRP, we calculated variable HRs ranging from 2 to 4 for NLR and 0.1 to 2.0 mg/dL for CRP in the test cohort, as shown in the Supplementary Figure 1. The smallest *p*-values were obtained at 3 (HR, 0.48; 95% CI, 0.31–0.73; *p =* 0.0004) for NLR and 1.0 mg/dL (HR, 0.44; 95% CI, 0.28–0.68; *p =* 0.0002) for CRP. We used these cut-off values and classified into NLR-high (≥ 3, *n =* 101) and -low (< 3, *n =* 78), and CRP-high (≥ 1 mg/dL, *n =* 62) and -low (< 1 mg/dL, *n =* 86).

### Clinicopathological characteristics of patients treated with bevacizumab plus paclitaxel according to NLR or CRP levels

There was no significant difference between NLR-high and -low groups in any clinicopathological factors. More patients had disease progression in the NLR-high (62.4%) group as compared with the NLR-low (46.2%, *p =* 0.083) group, although the difference was not statistically significant (Table 1). Similarly, no differences involving clinicopathological factors between CRP-high and -low groups were detected (Table 1).

**Table 1:** Clinicopathological characteristics of patients treated with bevacizumab and paclitaxel according to neutrophil-to-lymphocyte ratio (NLR) and c-reactive protein (CRP) in the test cohort

**Characteristics**	**NLR-high^a^ (*n* = 101)**	**NLR-low^a^ (*n* = 78)**	***p*-value **	**CRP-high^b^ (*n* = 62)**	**CRP-low^b^ (*n* = 86)**	***p*-value **
Age (year)						
Median (Range)	59 (35–83)	61 (34–79)	*p =* 0.288	59 (34–83)	61 (39–79)	*p =* 0.705
Menopausal status						
Pre-	16 (15.8%)	13 (16.7%)	*p =* 1.000	9 (14.5%)	16 (18.6%)	*p =* 0.801
Post-	84 (83.2%)	65 (83.3%)		53 (85.5%)	69 (80.2%)	
Unknown	1 (1.0%)	0 (0%)		0 (0%)	1 (1.2%)	
Estrogen receptor status						
Positive	75 (74.3%)	65 (83.3%)	*p =* 0.097	51 (82.3%)	69 (80.2%)	*p =* 0.507
Negative	26 (25.7%)	12 (16.0%)		10 (16.1%)	17 (19.8%)	
Unknown	0 (0%)	1 (1.3%)		1 (1.6%)	0 (0%)	
Progesterone receptor status						
Positive	57 (56.4%)	48 (61.5%)	*p =* 0.089	37 (59.7%)	54 (62.8%)	*p =* 0.884
Negative	44 (43.6%)	27 (34.6%)		24 (38.7%)	30 (34.9%)	
Unknown	0 (0%)	3 (3.9%)		1 (1.6%)	2 (2.3%)	
HER2 status						
Positive	7 (6.9%)	6 (7.7%)	*p =* 0.343	5 (8.1%)	3 (3.5%)	*p =* 0.468
Negative	94 (93.1%)	70 (89.7%)		56 (90.3%)	82 (95.4%)	
Unknown	0 (0%)	2 (2.6%)		1 (1.6%)	1 (1.2%)	
Ki67 expression levels						
< 25%	21 (20.8%)	12 (15.4%)	*p =* 0.479	10 (16.1%)	20 (23.3%)	*p =* 0.565
≥ 25%	33 (32.7%)	23 (29.5%)		16 (25.8%)	21 (24.4%)	
Unknown	47 (46.5%)	43 (55.1%)		36 (58.1%)	45 (52.3%)	
Subtype ^c^						
Luminal A	16 (15.8%)	10 (12.8%)	*p =* 0.163	7 (11.3%)	17 (19.8%)	*p =* 0.407
Luminal B	16 (15.8%)	15 (19.2%)		9 (14.5%)	13 (15.1%)	
Triple-negative	22 (21.8%)	7 (9.0%)		8 (12.9%)	15 (17.4%)	
HER2-positive	7 (6.9%)	6 (7.7%)		5 (8.1%)	3 (3.5%)	
Unknown	40 (39.6%)	40 (51.3%)		33 (53.2%)	38 (44.2%)	
Primary advanced or recurrence						
Primary advanced	38 (37.6%)	27 (34.6%)	*p =* 0.755	20 (32.3%)	33 (38.4%)	*p =* 0.490
Recurrence	63 (62.4%)	51 (65.3%)		42 (67.7%)	53 (61.6%)	
Metastatic sites						
Visceral	78 (77.2%)	66 (84.6%)	*p =* 0.125	52 (83.9%)	68 (79.1%)	*p =* 0.807
Non-visceral	23 (22.8%)	11 (14.1%)		10 (16.1%)	17 (19.8%)	
Unknown	0 (0%)	1 (1.3%)		0 (0%)	1 (1.2%)	
Prior endocrine therapy						
Yes	62 (61.4%)	56 (71.8%)	*p =* 0.156	41 (66.1%)	61 (70.9%)	*p =* 0.591
No	39 (38.6%)	22 (28.2%)		21 (33.9%)	25 (29.1%)	
Number of prior chemotherapy						
0 and 1	62 (61.4%)	43 (55.1%)	*p =* 0.406	38 (61.3%)	51 (59.3%)	*p =* 0.826
2 to 4	27 (26.7%)	28 (35.9%)		17 (27.4%)	27 (31.4%)	
5 and more	12 (11.9%)	7 (9.0%)		7 (11.3%)	8 (9.3%)	
Prior anthracycline therapy						
Yes	49 (48.5%)	46 (59.0%)	*p =* 0.177	28 (45.2%)	43 (50.0%)	*p =* 0.618
No	52 (51.5%)	32 (41.0%)		34 (54.8%)	43 (50.0%)	
Prior taxane therapy						
Yes	52 (51.5%)	39 (50.0%)	*p =* 0.881	30 (48.4%)	39 (45.4%)	*p =* 0.741
No	49 (48.5%)	39 (50.0%)		32 (51.6%)	47 (54.7%)	
Reason of treatment discontinuation						
Disease progression	63 (62.4%)	36 (46.2%)	*p =* 0.083	41 (66.1%)	42 (48.8%)	*p =* 0.151
Adverse events	19 (18.8%)	26 (33.3%)		12 (19.4%)	27 (31.4%)	
Ongoing	18 (17.8%)	15 (19.2%)		8 (12.9%)	16 (18.6%)	
Others	1 (1.0%)	1 (1.3%)		1 (1.6%)	1 (1.2%)	

^a^high: ≥ 3, low: < 3. ^b^high: ≥ 1.0 mg/dL, low: < 1.0mg/dL. ^c^Luminal A, estrogen receptor (ER)-positive/HER2-negative and Ki67 < 25%; Luminal B, ER-positive/HER2-negative and Ki67 ≥ 25%; TN, ER-negative/HER2-negative; HER2, HER2-positive; Unknown, ER-positive/HER2-negative and Ki67 unknown.

### Kaplan-Meier plots of patients according to NLR, CRP, and ALC levels for PFS or OS in the test cohort

**Figure 1:**
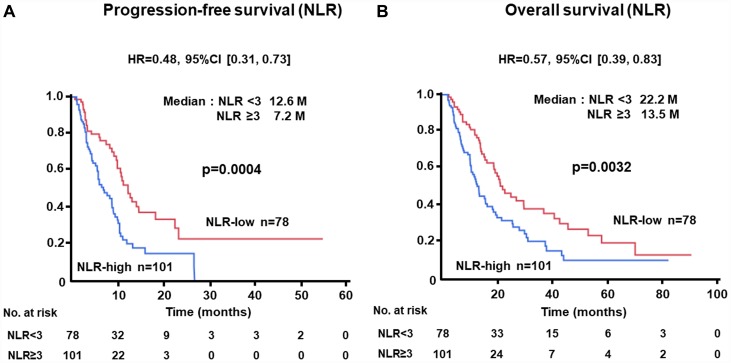
(**A**) Progression-free survival (PFS) and (**B**) overall survival (OS) of patients grouped by neutrophil-to-lymphocyte ratio (NLR) < 3 or ≥ 3 groups in the test cohort.

**Figure 2:**
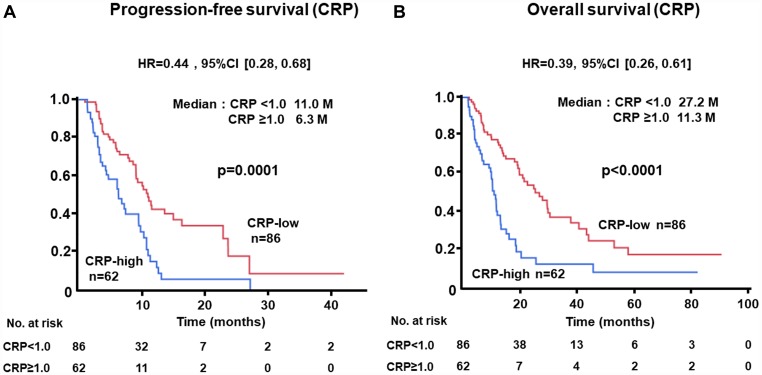
(**A**) Progression-free survival (PFS) and (**B**) overall survival (OS) of patients grouped by c-reactive protein (CRP) < 1.0 mg/dL or ≥ 1.0 mg/dL groups in the test cohort.

**Figure 3:**
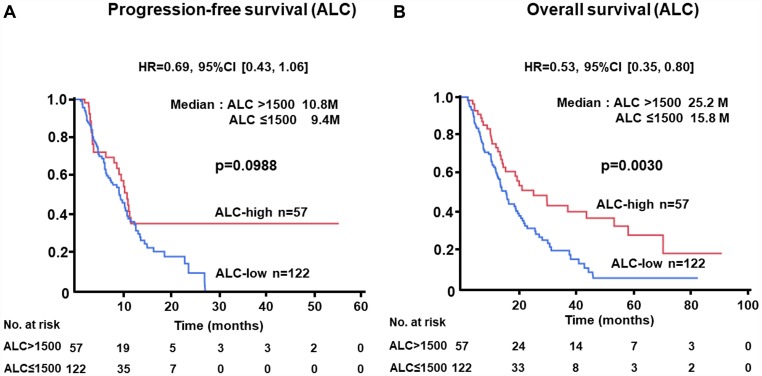
(**A**) Progression-free survival (PFS) and (**B**) overall survival (OS) of patients grouped by absolute lymphocyte count (ALC) > 1500 /μL or ≤ 1500 /μL groups in the test cohort.

The PFS of patients with low levels of NLR at baseline was significantly longer than that of cases with high levels (median, 12.6 vs. 7.2 months; HR, 0.48; 95% CI, 0.31–0.73; *p =* 0.0004) (Figure 1A). Similarly, longer OS was recognized in the NLR-low group than in the NLR-high subjects (median, 22.2 vs. 13.5 months; HR, 0.57; 95% CI, 0.39–0.83; *p =* 0.0032) (Figure 1B). Longer PFS (median, 11.0 vs. 6.3 months; HR, 0.44; 95% CI, 0.28–0.68; *p =* 0.0001) and OS (median, 27.2 vs. 11.3 months; HR, 0.39; 95% CI, 0.26–0.61; *p <* 0.0001) were demonstrated in patients with low levels of CRP as compared with those with higher levels (Figure 2A, 2B). There was no significant difference in PFS between ALC-high and -low groups (median, 10.8 vs. 9.4 months; HR, 0.69; 95% CI, 0.43–1.06; *p =* 0.0988); in contrast, OS of patients with high levels of ALC was significantly better than those with low levels (median, 25.2 vs. 15.8 months; HR, 0.53; 95% CI, 0.35–0.80; *p =* 0.0030) (Figure 3A, 3B).

### Subgroup analyses of PFS according to clinical factors

**Figure 4:**
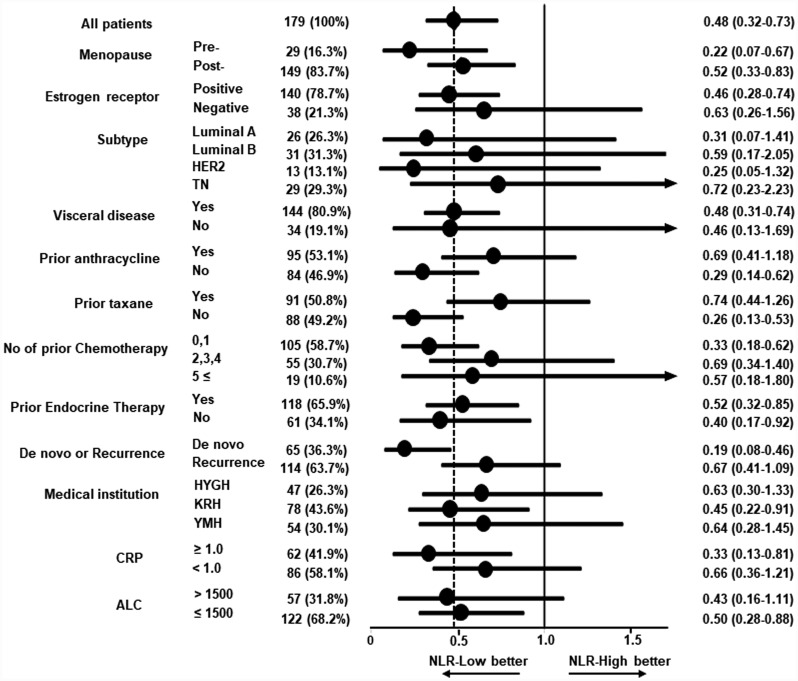
Forest plot for subgroup analysis of progression-free survival among patients treated with bevacizumab plus paclitaxel. Hazard ratios (HRs) and 95% confidence intervals (CIs) in several subgroups are shown. The HR of 0.48 for all patients is shown by the dashed line. Progression-free survival was better in the low neutrophil-to-lymphocyte ratio (NLR) group across all subgroups. Subtypes: Luminal A, estrogen receptor (ER)-positive/HER2-negative and Ki67 < 25%; Luminal B, ER-positive/HER2-negative and Ki67 ≥ 25%; HER2, HER2-positive; TN, ER-negative/HER2-negative. Medical institutions: HYGH, Hyogo College of Medicine; KRN, Kansai Rosai Hospital; YMH, Yao Municipal Hospital. CRP: c-reactive protein. ALC: absolute lymphocyte count.

Consistently improved PFS in patients with NLR-low was recognized across all subgroups (Figure 4). Favorable HRs for PFS were obtained irrespective of subtype (HR, 0.31; 95% CI, 0.07–1.41 for luminal A; HR, 0.59; 95% CI, 0.17–2.05 for luminal B; HR, 0.25; 95% CI, 0.05–1.32 for HER2; HR, 0.72; 95% CI, 0.23–2.23 for triple negative (TN)), visceral disease (HR, 0.48; 95% CI, 0.31–0.74 for visceral disease; HR, 0.46; 95% CI, 0.13–1.69 for non-visceral disease), and prior number of chemotherapy treatments (HR, 0.33; 95% CI, 0.18–0.62 for 0 or 1 prior chemotherapy; HR, 0.69; 95% CI, 0.34–1.40 for 2 to 4 prior chemotherapies; HR, 0.57; 95% CI, 0.18–1.80 for 5 and more prior chemotherapies). In addition, better PFS in the NLR-low group was consistently recognized in the CRP-high (HR, 0.33; 95% CI, 0.13–0.81) and -low (HR, 0.66; 95% CI, 0.36–1.21) groups and ALC-high (HR, 0.43; 95% CI, 0.16–1.11) and -low (HR, 0.50; 95% CI, 0.28–0.88) groups.

### Univariable and multivariable analyses for PFS and OS

By univariable analysis, subtypes, the number of prior chemotherapy treatments, prior taxane use, NLR, and CRP levels were significant predictive factors for PFS (Table 2). Multivariable analysis of these factors, subtypes (HR, 2.62; 95% CI, 1.06–6.61; *p =* 0.036 for TN and HR, 4.08; 95% CI, 1.12–13.31; *p =* 0.035 for HER2), NLR (HR, 0.34; 95% CI, 0.14–0.77; *p =* 0.008), and CRP (HR, 0.35; 95% CI, 0.16–0.74; *p =* 0.006) were independently and significantly associated with PFS. Two subtypes (HR, 3.27; 95% CI, 1.20–9.58; *p =* 0.020 for luminal B; and HR, 5.52; 95% CI, 1.35–21.28; *p =* 0.019 for HER2) were significantly and independently associated with OS by multivariable analysis (Table 3). Using multivariable analysis, we found that CRP trended toward significance (HR, 0.44, 95% CI, 0.20–1.01; *p =* 0.052).

**Table 2:** Univariable and multivariable analyses of progression-free survival for patients treated with bevacizumab and paclitaxel in the test cohort

	**Univariable analysis**			**Multivariable analysis**	
	***n***	**HR (95% CI)^a^**	***p*-value **	**HR (95% CI)^a^**	***p*-value **
Menopausal status					
Premenopausal	29	1.00			
Postmenopausal	149	0.79 (0.48-1.37)	0.382		
Subtype ^b^					
Luminal A	26	1.00		1.00	
Luminal B	31	1.25 (0.54-2.80)	0.596	1.55 (0.59-3.91)	0.362
Triple-negative	29	2.36 (1.17-4.92)	0.017	2.62 (1.06-6.61)	0.036
HER2-positive	13	2.69 (1.10-6.28)	0.031	4.08 (1.12-13.31)	0.035
Primary advanced or recurrence					
Primary advanced	65	1.00			
Recurrence	114	1.27 (0.84-1.97)	0.258		
Metastatic sites					
Non-visceral	144	1.00			
Visceral	34	1.19 (0.69-2.25)	0.544		
Number of prior chemotherapy					
0 and 1	105	1.00		1.00	
2 to 4	55	1.18 (0.75-1.82)	0.467	0.78 (0.28-2.03)	0.614
5 and more	19	1.94 (1.05-3.38)	0.035	0.59 (0.15-1.80)	0.374
Prior anthracycline therapy					
No	84	1.00			
Yes	95	1.17 (0.79-1.76)	0.435		
Prior taxane therapy					
No	88	1.00		1.00	
Yes	91	1.58 (1.06-2.38)	0.024	1.36 (0.63-2.93)	0.435
Prior endocrine therapy					
No	61	1.00			
Yes	118	0.88 (0.58-1.37)	0.565		
Neutrophil- to-lymphocyte ratio ^c^					
High	101	1.00		1.00	
Low	78	0.48 (0.31-0.73)	0.0004	0.34 (0.14-0.77)	0.008
C-reactive protein ^d^					
High	62	1.00		1.00	
Low	86	0.49 (0.28-0.68)	0.0002	0.35 (0.16-0.74)	0.006

^a^Hazard ratio (95% confidence interval). ^b^Luminal A, estrogen receptor (ER)-positive/HER2-negative and Ki67 < 25%; Luminal B, ER-positive/HER2-negative and Ki67 ≥ 25%; TN, ER-negative/HER2-negative; HER2, HER2-positive; Unknown, ER-positive/HER2-negative and Ki67 unknow. ^c^high: ≥ 3, low: < 3. ^d^high: ≥ 1.0 mg/dL, low: < 1.0 mg/dL.

**Table 3:** Univariable and multivariable analyses of overall survival for patients treated with bevacizumab and paclitaxel in the test cohort

	***n***	**Univariable analysis**		**Multivariable analysis**	
		**HR (95% CI)^a^**	***p*-value **	**HR (95% CI)^a^**	***p*-value **
Menopausal status					
Premenopausal	29	1.00			
Postmenopausal	149	0.73 (0.46–1.22)	0.214		
Subtype ^b^					
Luminal A	26	1.00		1.00	
Luminal B	31	2.05 (0.97–4.61)	0.061	3.27 (1.20–9.58)	0.020
Triple-negative	29	2.47 (1.17–5.55)	0.018	2.30 (0.86–6.60)	0.097
HER2-positive	13	3.51 (1.39–8.80)	0.009	5.52 (1.35–21.28)	0.019
Primary advanced or recurrence					
Primary advanced	65	1.00		1.00	
Recurrence	114	2.05 (1.37–3.15)	0.0004	1.35 (0.54–3,23)	0.517
Metastatic sites					
Non-visceral	144	1.00			
Visceral	34	1.51 (0.95–2.52)	0.086		
Number of prior chemotherapy					
0 and 1	105	1.00		1.00	
2 to 4	55	1.83 (1.22–2.72)	0.004	1.03 (0.40–2.61)	0.953
5 and more	19	2.40 (1.33–4.10)	0.005	2.21 (0.67–6.65)	0.185
Prior anthracycline therapy					
No	84	1.00		1.00	
Yes	95	2.04 (1.39–3.05)	0.0003	0.99 (0.39–2.61)	0.990
Prior taxane therapy					
No	88	1.00		1.00	
Yes	91	2.42 (1.66–3.56)	<0.0001	1.73 (0.64–4.83)	0.284
Prior endocrine therapy					
No	61	1.00			
Yes	118	1.39 (0.94–2.12)	0.100		
Neutrophil- to-lymphocyte ratio^c^					
High	101	1.00		1.00	
Low	78	0.57 (0.39–0.83)	0.0033	0.57 (0.24–1.30)	0.186
C-reactive protein^d^					
High	62	1.00		1.00	
Low	86	0.39 (0.26–0.61)	<0.0001	0.44 (0.20–1.01)	0.052

^a^Hazard ratio (95% confidence interval). ^b^Luminal A, estrogen receptor (ER)-positive/HER2-negative and Ki67 < 25%; Luminal B, ER-positive/HER2-negative and Ki67 ≥ 25%; TN, ER-negative/HER2-negative; HER2, HER2-positive; Unknown, ER-positive/HER2-negative and Ki67 unknow. ^c^high: ≥ 3, low: < 3. ^d^high: ≥ 1.0 mg/dL, low: < 1.0 mg/dL.

### PFS and OS of patients divided into four groups based on NLR and CRP levels

**Figure 5:**
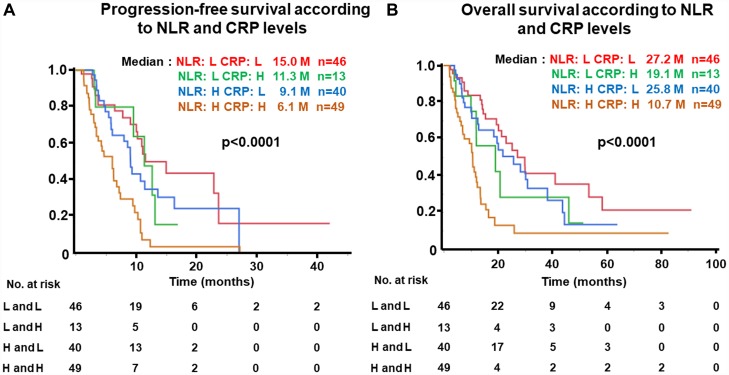
(**A**) Progression-free survival (PFS) and (**B**) Overall survival (OS) of patients grouped by neutrophil-to-lymphocyte ratio (NLR) and c-reactive protein (CRP) levels. NLR-high ≥ 3, NLR-low, < 3; CRP-high, ≥ 1.0 mg/dL, CRP-low, < 1.0 mg/dL in the test cohort.

Kaplan-Meier analysis for PFS was performed according to four groups divided by combination of NLR and CRP levels, as shown in Figure 5. PFS of patients with NLR-low and CRP-low was highest (median, 15.0 months) and lowest in the NLR-high and CRP-high group (6.1 months, *p <* 0.0001) (Figure 5A). Similarly, OS was significantly associated with NLR and CRP levels (NLR-low and CRP-low, 27.2 vs. NLR-high and CRP-high, 10.7 months, *p <* 0.0001) (Figure 5B).

### PFS and OS of patients according to NLR, CRP, or ALC levels in the validation cohort

We analyzed the prognostic significance of NLR, ALC, and CRP levels in the validation cohort using the cut-off values defined in the test cohort. The PFS of patients with NLR-low (median, 8.9 months) was significantly longer than those with NLR-high (median, 6.1 months; HR, 0.47; 95% CI, 0.22–0.97; *p =* 0.0344) (Supplementary Figure 2A). As for OS, in patients with NLR-low (median, 36.8 months), OS was significantly longer than those with NLR-high (median, 18.4 months; HR, 0.43; 95% CI, 0.20–0.91; *p =* 0.0233) (Supplementary Figure 2B). Similarly, in patients with low levels of CRP, both PFS and OS were significantly longer than in patients with high levels (12.4 vs 3.5 months; HR, 0.22; 95% CI, 0.10–0.47; *p <* 0.0001; and 35.4 vs 7.4 months; HR, 0.24; 95% CI, 0.11–0.54; *p =* 0.0001, respectively) (Supplementary Figure 3). For ALC, there was a significant association between ALC levels and PFS (*p =* 0.0247), but no significant association was found in OS (*p =* 0.0841) (Supplementary Figure 4). When we combined NLR and CRP, the longest PFS and OS was observed in the NLR-low and CRP-low group (median 17.3 months and not reached, respectively) (Supplementary Figure 5). On the contrary, PFS and OS of patients with NLR-high and CRP-high (median 4.0 and 7.9 months, respectively) and NLR-low and CRP-high (median 3.3 and 7.4 months, respectively) were shorter.

## DISCUSSION

In this study, significant improvement after bevacizumab plus paclitaxel therapy was obtained in patients with low levels of NLR and CRP at baseline in terms of OS, as well as PFS as compared with those with high levels in both the test and validation cohorts. On the contrary, consistent significant associations between ALC and PFS, or between ALC and OS, were not obtained - neither in the test cohort nor the validation cohort. In addition, low levels of NLR and CRP were significantly and independently associated with longer PFS. These data indicated a usefulness of NLR and CRP as predictive markers of bevacizumab in addition to paclitaxel for MBC. Gampenrieder *et al*. constructed a DNA methylation signature (9 gene signature) that predicts bevacizumab efficacy in MBC (HR, 0.18; 95% CI, 0.01-NA, *p <* 0.001 for PFS; and HR, 0.24; 95% CI, 0.07–0.38, *p <* 0.001 for OS) [15]. Furthermore, predicting tools (G and GC models) for bevacizumab plus paclitaxel based on angiogenesis-related profiling have been demonstrated by Mendiola *et al*. [16]. These G- and GC-models identified subgroups with improved PFS (HR, 2.57 and 4.04, respectively) and OS (HR, 3.29 and 3.43, respectively).

In addition to these predictive models based on factors at baseline, the usefulness as a predictor of early changes to factors associated with angiogenesis or hypoxia after commencement of bevacizumab-based therapy was reported. High levels of carbonic anhydrase (CA9) after a cycle of treatment were significantly associated with longer PFS (*p <* 0.001) and OS (*p <* 0.001) as compared with low levels of CA9 increase [17]. Since CA9 is induced under hypoxic conditions by hypoxia inducible factor-1 (HIF1) [18], alteration of the microenvironment, including the hypoxic condition, by an angiogenesis inhibitor may be associated with its efficacy. In line with this concept, vessel architectural imaging (VAI) was established for identification of patients expected to respond to anti-angiogenic therapy [19]. This technique was developed based on magnetic resonance imaging (MRI) for monitoring of vessel functions including hemodynamic status, vascular structure, and oxygen saturation (SO_2_) levels. Among patients with recurrent glioblastomas treated with anti-angiogenic cediranib, treatment was effective in patients who achieved more competent microcirculation by a relative increase in vessel vortex direction in the tumor center with reduced vessel calibers accompanied by upregulation of SO_2_. These data suggested that efficacy of angiogenesis inhibitors is mediated through not only reduction of neovasculature, but also normalization of tumor vessels and improvement in hypoxic conditions in the tumor.

It is reported that normalization of tumor vessels by inhibiting angiopoietin (Ang) 2, which is an enhancer of angiogenesis, and Tie2 activation, which induces vessel stabilization, leads to enhancement of drug delivery and production of favorable tumor microenvironments in a mouse model [20]. Interestingly, favorable changes to immune cell infiltration were observed by normalization of tumor vessels in this glioma model. Furthermore, targeting of tumor vessels using anti-VEGFR-2 antibody with lower doses inducing vascular normalization, but not high doses, resulted in reprograming the immune suppressive microenvironment toward the potentiation of immune active status [21]. Vascular normalization by anti-angiogenic therapy, therefore, seems to be clinically important in terms of immune reactions against cancer cells, especially mediated through T lymphocyte infiltration [22].

The precise mechanisms why NLR and CRP, but not ALC levels, are associated with PFS of patients treated with bevacizumab plus paclitaxel have yet to be resolved. According to Chen *et al.*’s study, high levels of NLR were significantly associated with high levels of circulating inflammatory cytokines, angiogenic cytokines, and epidermal growth factor ligands [23]. We demonstrated that the difference in median OS (8.7 months) between NLR-low and -high groups was higher than that in PFS (5.4 months). Similarly, increased difference in the OS (15.9 months) than in PFS (4.7 months) was recognized between CRP-low and -high groups. These data may represent a possible involvement of cancer immunity modified by bevacizumab. Since high levels of NLR were significantly associated with low levels of natural killer (NK) cell activity in healthy individuals [24] and high levels of peripheral MDSC in breast cancer patients [25], NLR seems to be a surrogate for immune status against cancer cells, at least in part. Considering the function of bevacizumab, which reduces VEGF activity in addition to vascular normalization, promotes immune reactions mediated through several factors including reduction of TAM, Tregs, or MDSCs, and maturation of DCs [13], such immune modulation leads to a favorable prognosis especially in terms of OS. Since patients with low levels of NLR or CRP had greater benefit from bevacizumab as compared with those with high levels, these markers may indicate that the microenvironment in breast cancer is likely to afford sufficient efficacy induced by bevacizumab. We failed to demonstrate consistent positive associations between ALC levels and longer PFS or OS, which may indicate a predictive significance not of ALC, but of NLR and CRP for bevacizumab efficacy.

CRP is well known as an inflammatory marker, and high levels of CRP significantly are associated with worse OS in MBC [26]. In colorectal cancer, CRP levels are significantly positively associated with VEGF-A (*r* = 0.23, *p <* 0.0001) and Ang2 (*r* = 0.43, *p <* 0.001) [27]. On the basis of these results, the association between CRP levels and bevacizumab efficacy may be in line with that of angiogenesis. Since there was no significant association between NLR and CRP levels (data not shown), and NLR and CRP are independent factors for PFS by multivariable analysis, we believe these markers have roles involving different mechanisms in bevacizumab’s effects. The limitation of this study is that these results were obtained from retrospective real-world data consisting of several treatment lines of bevacizumab for locally advanced or MBC. Furthermore, detailed mechanisms involving NLR and CRP for predicting efficacy of bevacizumab remain unknown. Another limitation was the cut-off values of NLR and CRP. In the present study, we obtained optimal cut-off values of NLR and CRP at 3.0 and 1.0 mg/dL, respectively, based on the data from the test cohort. Since the significance of these cut-off values was confirmed in the validation cohort using different automatic analyzers for NLR and CRP, the issue of whether these cut-off values are widely applicable in clinical settings or not still remains unknown. We need to confirm these data prospectively in future studies with larger numbers of patients.

In conclusion, we have shown that low levels of NLR and CRP are significantly associated with improved PFS and OS in advanced breast cancer treated with bevacizumab plus paclitaxel. The data obtained here seem to be useful not only for selecting patients who have benefit from bevacizumab-based therapy, but also for identifying subgroups for combination therapy with bevacizumab and immune checkpoint inhibitors. This is the first study to demonstrate the predictive utility of NLR and CRP for bevacizumab plus paclitaxel therapy in breast cancer to the best of our knowledge.

## MATERIALS AND METHODS

### Patient eligibility

A total of 202 patients treated with bevacizumab plus paclitaxel were retrospectively collected from three institutes (Hyogo College of Medicine, *n =* 60; Kansai Rosai Hospital, *n =* 87; Yao Municipal Hospital, *n =* 55) in the test cohort. Patients were excluded if they: were treated for less than one cycle (*n =* 18), had no blood data at baseline (*n =* 2), or were males with breast cancer (n =3). In the end, 179 patients (Hyogo College of Medicine, *n =* 47; Kansai Rosai Hospital, *n =* 78; Yao Municipal Hospital, *n =* 54) were enrolled for this part of the study (Supplementary Figure 6). In the validation cohort, 63 patients were recruited from two new institutes (Kobe City Medical Center, *n =* 40 and Amagasaki General Medical Center, *n =* 23). Since six patients were excluded due to having less than one cycle of treatment, a total of 57 patients (Kobe City Medical Center, *n =* 35 and Amagasaki General Medical Center, *n =* 22) were analyzed in the validation cohort (Supplementary Figure 6). Primary breast cancer was confirmed by histology, and locally advanced state or metastases were diagnosed radiographically. HER2-positive breast cancers were eligible for treatment with bevacizumab plus paclitaxel without anti-HER2 therapy. Subtypes were classified as Luminal A, Luminal B, TN, and HER2. Accordingly, the TN (*n =* 29) and HER2 (*n =* 13) subtypes were defined as ER-negative/HER2-negative and HER2-positive, respectively. ER-positive/HER2-negative breast cancers were further divided on the basis of Ki67 expression levels as follows: Luminal A, Ki67 < 25%, *n =* 26; Luminal B, Ki67 ≥ 25%, *n =* 31. In the 80 ER-positive/HER2-negative breast cancers, there was no data on Ki67 expression levels.

### Bevacizumab plus paclitaxel schedule and evaluation of outcome

Bevacizumab 10 mg/kg on days 1 and 15 and paclitaxel 80 or 90 mg/m^2^ on days 1, 8, and 15 of each 28-day cycle were administered intravenously. For patients with adverse events, the dose of paclitaxel was reduced to 60 or 40 mg/m^2^ and paclitaxel was omitted when decreased neutrocyte counts or sever adverse events were noted. After the initial combination therapy, paclitaxel administration was stopped and only nine patients continued with bevacizumab. Bevacizumab with or without paclitaxel was discontinued due to disease progression and intolerable adverse events in 99 and 45 patients, respectively, and treatment was ongoing in 33 patients in the test cohort (Table 1). In the validation cohort, bevacizumab with or without paclitaxel was discontinued due to disease progression, intolerable adverse events, and other reasons in 32, 13, and three patients, respectively, and treatment was ongoing in nine patients. PFS was defined from the start of treatment until disease progression or death due to any reason, and OS was defined from the start of treatment until death due to any reason.

### Measurements of NLR, ALC, and CRP

Neutrophil and lymphocyte counts were measured automatically with Sysmex hematology analyzers XN-9000, XN-1000, and XE-5000 (Sysmex Corporation, Kobe, Japan) in Hyogo College of Medicine, Kansai Rosai Hospital, and Yao Municipal Hospital, respectively. The NLR of each patient was calculated by dividing the number of neutrophils by the number of lymphocytes. CRP was measured with LABOSPECT008 (Hitachi High-Technologies Corporation, Tokyo Japan) in Hyogo College of Medicine, cobas8000 (c702) (Roche Diagnostics K. K., Japan) in Kansai Rosai Hospital, and N-assay CRP-S (NITTOBO Medical Co., Ltd, Japan) in Yao Municipal Hospital. The normal range of CRP was ≤ 0.3 mg/dL. In the validation set, neutrophil and lymphocyte counts were measured automatically with Sysmex hematology analyzers XN-3100 and XN-9000 at the Kobe City Medical Center and Amagasaki General Medical Center, respectively. To measure CRP, we used a LABOSPEC 008l (Hitachi High-Technologies Corporation) and a TBA2000FR (CANON MEDICAL SYSTEMS LTD, Tochigi, Japan) in Kobe City Medical Center and Amagasaki General Medical Center, respectively. Blood was taken on the same day as the 1^st^ administration before the start of treatment. This study was approved by the ethics committee of the Hyogo College of Medicine (No. 1969) and conducted in accordance with the Declaration of Helsinki. As this study collected only retrospective clinical data and offered no risk to the participants, we did not ask for their written informed consent.

### Statistical analysis

Kaplan-Meier plots and log-rank tests of PFS or OS were applied for the different groups. The relationships between clinicopathological characteristics and NLR or CRP levels were calculated by Fisher’s exact test or the Wilcoxon rank-sum test. Univariate and multivariate analyses for PFS or OS were performed using a Cox proportional-hazards model to obtain the HR and 95% CI. Statistical significance was set at *p <* 0.05 and the statistical calculations were performed using JMP Pro 11 (SAS Institute Inc., Cary, NC, USA).

## SUPPLEMENTARY MATERIALS



## Abbreviations

MBC: metastatic breast cancer
VEGF: vascular endothelial growth factor
VEGFR: vascular endothelial growth factor receptor
PFS: progression-free survival
HR: hazard ratio
CI: confidence interval
OS: overall survival
HER 2: human epidermal growth factor receptor 2
Treg: regulatory T cell
DC: dendritic cell
TAM: tumor-associated macrophage
MDSC: myeloid-derived suppressor cell
NLR: neutrophil-to-lymphocyte ratio
ALC: absolute lymphocyte count
CRP: c-reactive protein
TN: triple negative
CA9: carbonic anhydrase
HIF1: hypoxia inducible factor-1
VAI: vessel architectural imaging
MRI: magnetic resonance imaging; SO_2_: oxygen saturation
Ang: angiopoietin
NK: natural killer
