# Supplementary material for: Baseline neutrophil-to-lymphocyte ratio and c-reactive protein predict efficacy of treatment with bevacizumab plus paclitaxel for locally advanced or metastatic breast cancer
Source: Oncotarget. 2020 Jan 7;11(1):86–98. doi: 10.18632/oncotarget.27423 (PMC6967770; doi:10.18632/oncotarget.27423)
Supplement: Supplementary file 1 [file oncotarget-11-86-s001.pdf]

Baseline neutrophil-to-lymphocyte ratio and c-reactive protein predict efficacy of treatment with bevacizumab plus paclitaxel for locally advanced or metastatic breast cancer

SUPPLEMENTARY MATERIALS

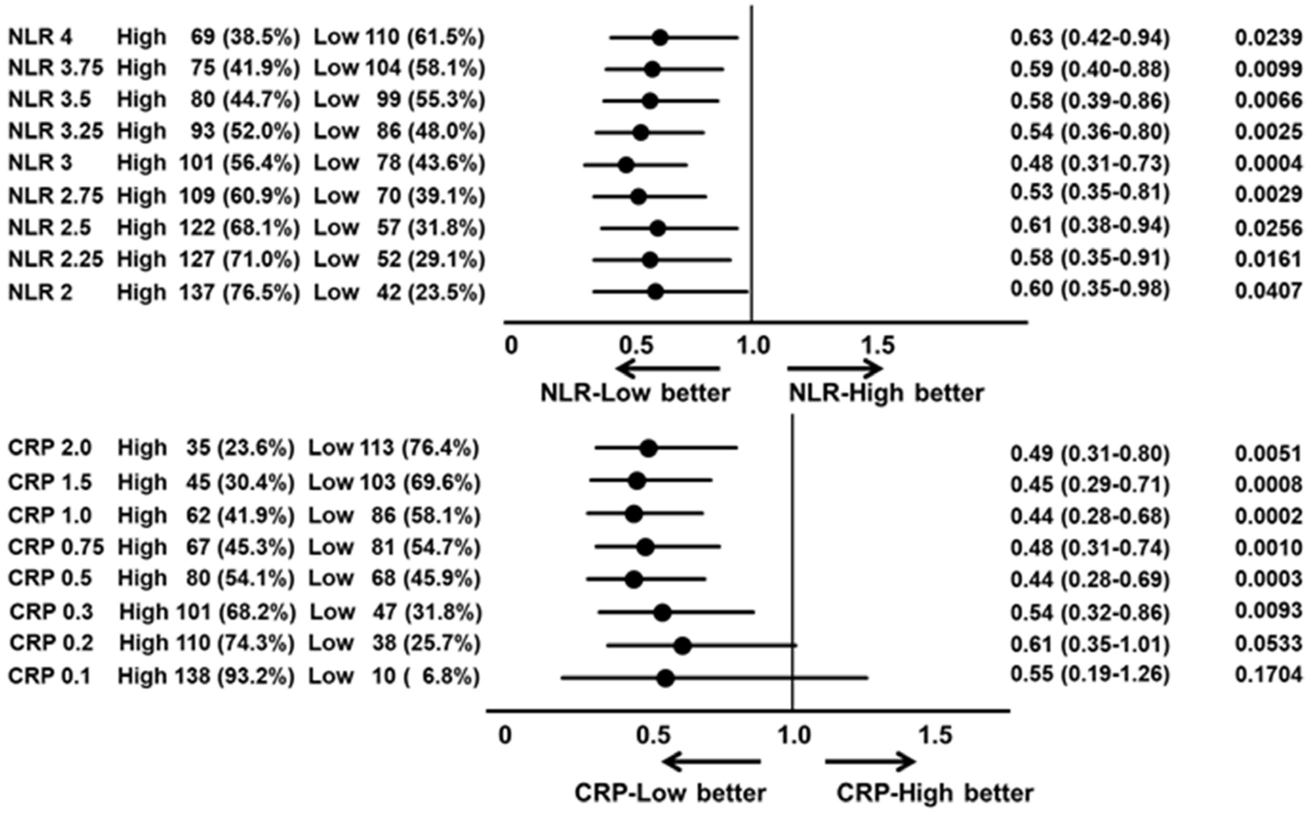

Supplementary Figure 1: Hazard ratios (HRs) and 95% confidence intervals (CIs) of progression-free survival of patients grouped by different cut-off values of neutrophil-to-lymphocyte ratio (NLR) from 2.0 to 4.0 and c-reactive protein (CRP) from 0.1 mg/dL to 2.0 mg/dL.

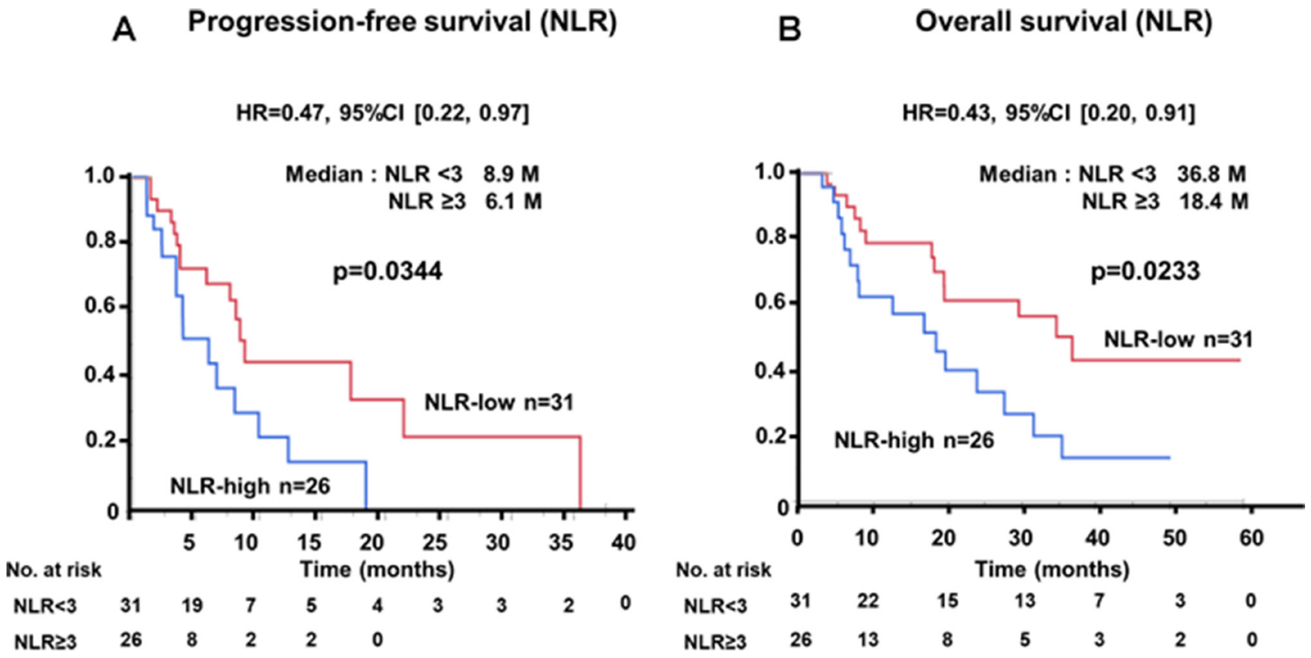

Supplementary Figure 2: (**A**) Progression-free survival (PFS) and (**B**) overall survival (OS) of patients grouped by neutrophil-to-lymphocyte ratio (NLR) < 3 or ≥ 3 in the validation cohort.

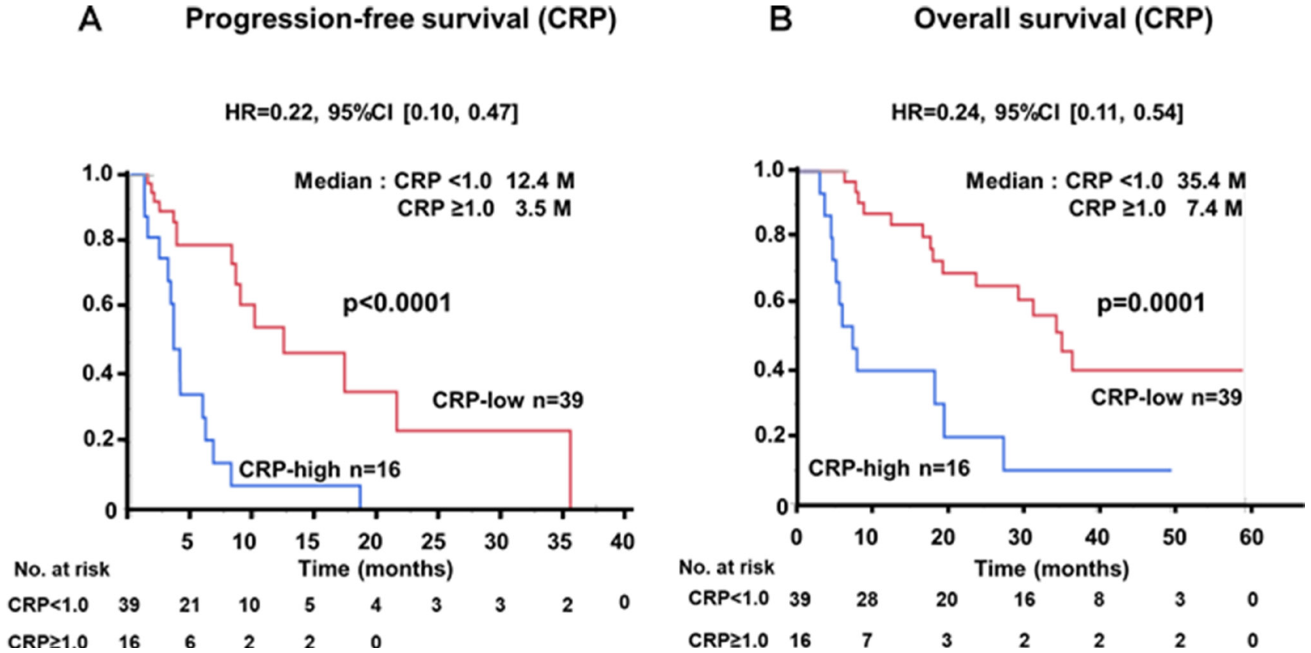

Supplementary Figure 3: (**A**) Progression-free survival (PFS) and (**B**) overall survival (OS) of patients grouped by c-reactive protein (CRP) < 1.0 mg/dL or ≥ 1.0 mg/dL in the validation cohort.

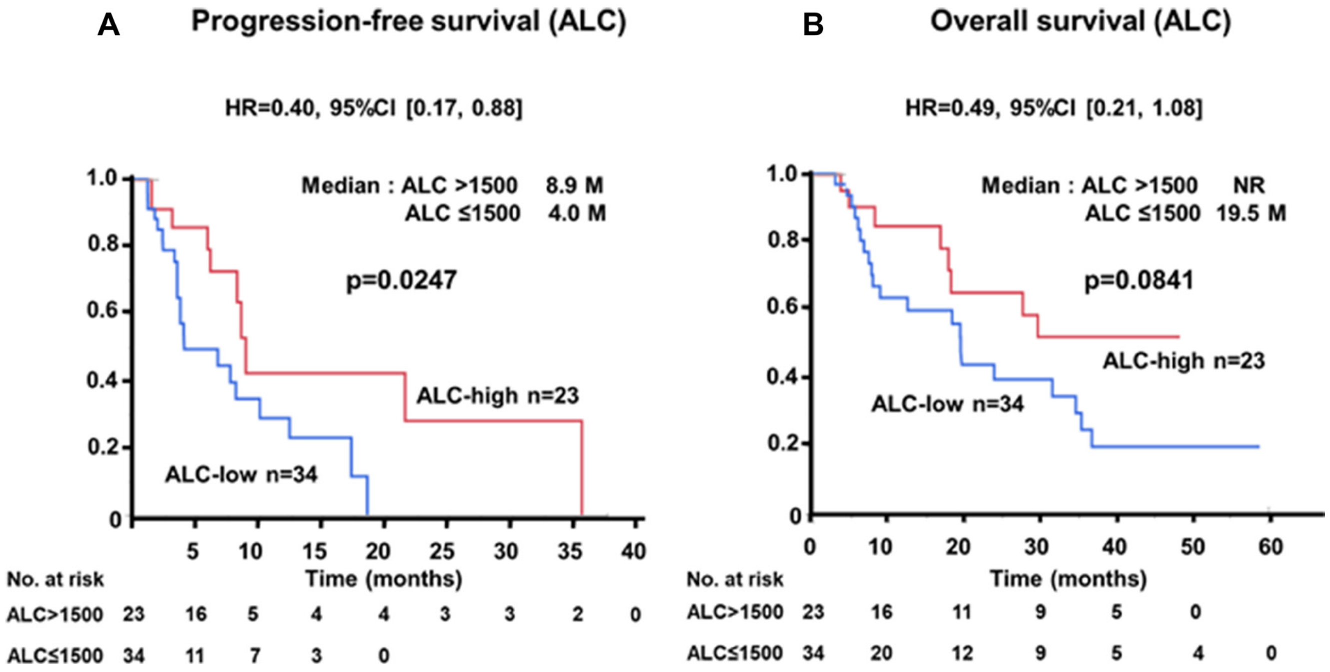

Supplementary Figure 4: (**A**) Progression-free survival (PFS) and (**B**) overall survival (OS) of patients grouped by absolute lymphocyte count (ALC) > 1500 /μL or ≤ 1500 /μL groups in the validation cohort.

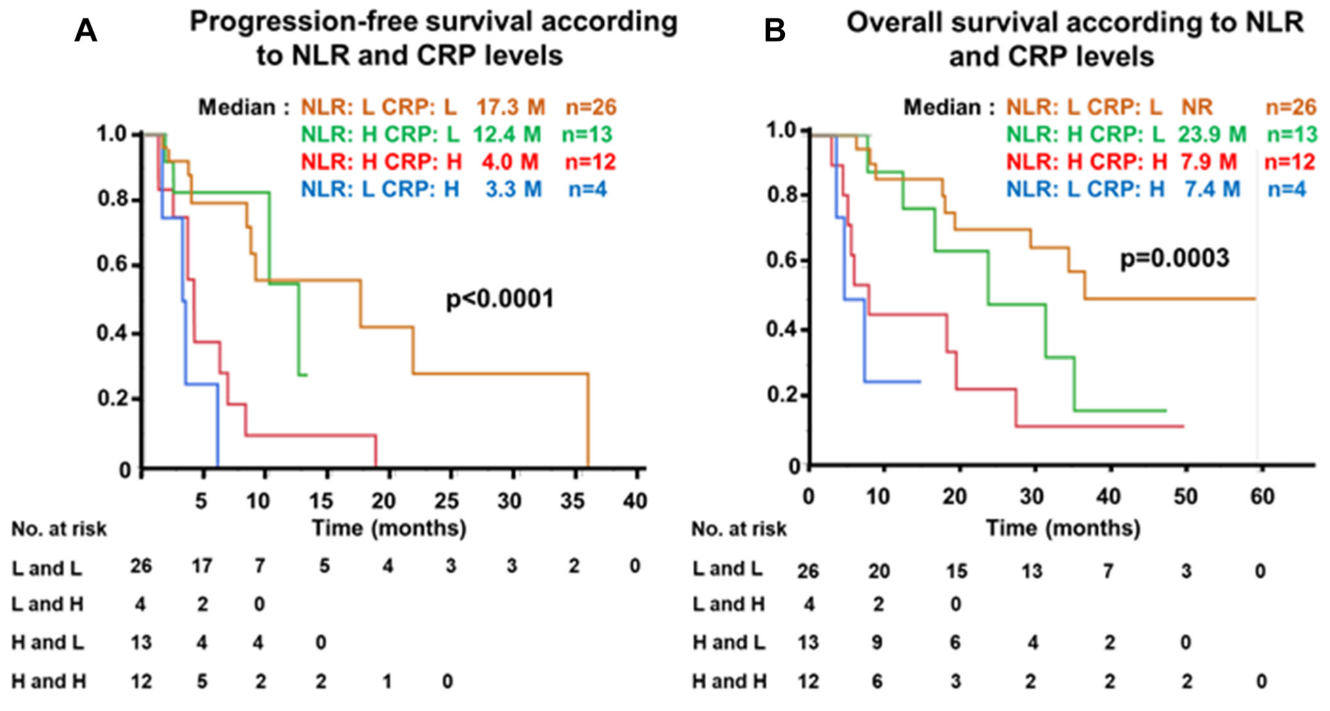

Supplementary Figure 5: (**A**) Progression-free survival (PFS) and (**B**) Overall survival (OS) of patients grouped by neutrophil-to-lymphocyte ratio (NLR) and c-reactive protein (CRP) levels. NLR-high ≥ 3, NLR-low, < 3; CRP-high, ≥ 1.0 mg/dL, CRP-low, < 1.0 mg/dL in the validation cohort.

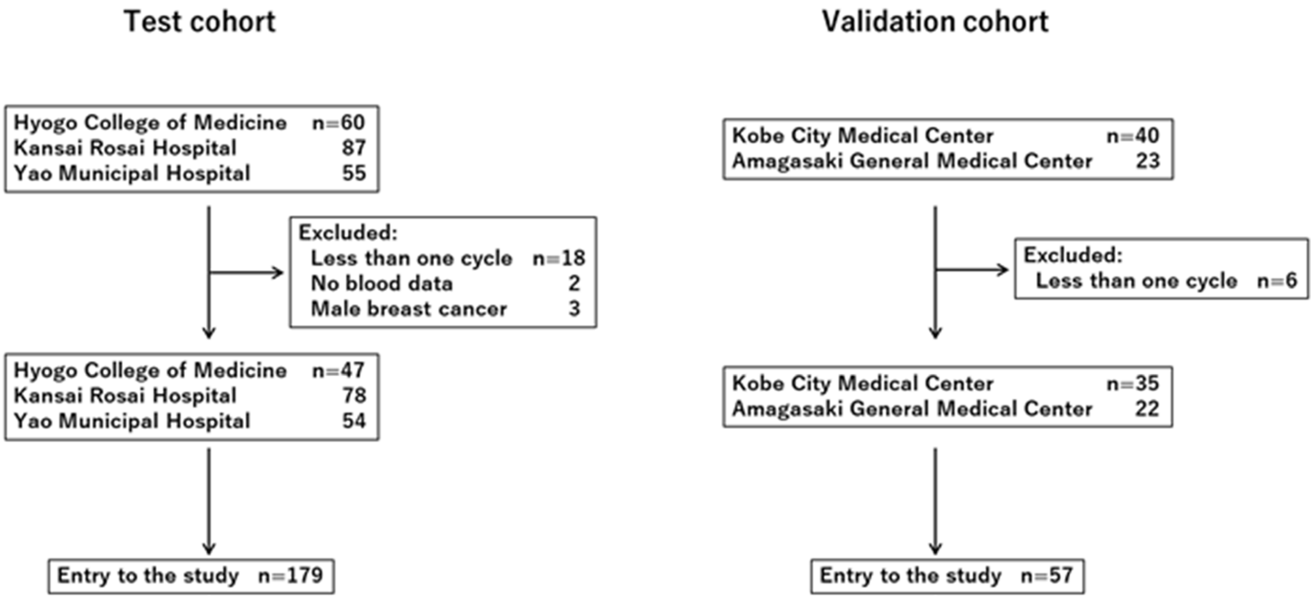

Supplementary Figure 6: Study diagram of patient eligibility in the test cohort and validation cohort. Patients who had less than one cycle of treatment, who had no blood data at baseline, and who were males with breast cancer were excluded.
